# Connectedness of COVID vaccination with economic policy uncertainty, oil, bonds, and sectoral equity markets: evidence from the US

**DOI:** 10.1007/s10479-023-05267-9

**Published:** 2023-03-22

**Authors:** Imran Yousaf, Saba Qureshi, Fiza Qureshi, Mariya Gubareva

**Affiliations:** 1 College of Business and Public Management, Wenzhou-Kean University, Wenzhou, China; 2 Institute of Business Administration, University of Sindh, Jamshoro, Pakistan; 3 Southampton Malaysia Business School, University of Southampton Malaysia, Iskandar Puteri, Malaysia; 4 ISEG – Lisbon School of Economics and Management, Universidade de Lisboa, Av. Miguel Lupi, 20, 1249-078 Lisbon, Portugal; 5 SOCIUS/CSG - Research in Social Sciences and Management, Rua Miguel Lupi, 20, 1249-078 Lisbon, Portugal

**Keywords:** COVID-19 vaccination, Economic policy uncertainty, Crude oil, Sectoral equity markets, Corporate and Treasury bonds, Wavelet approach, F36, N2, N7, P34, Q4

## Abstract

We examine the connectedness of the COVID vaccination with the economic policy uncertainty, oil, bonds, and sectoral equity markets in the US within time and frequency domain. The wavelet-based findings show the positive impact of COVID vaccination on the oil and sector indices over various frequency scales and periods. The vaccination is evidenced to lead the oil and sectoral equity markets. More specifically, we document strong connectedness of vaccinations with communication services, financials, health care, industrials, information technology (IT) and real estate equity sectors. However, weak interactions exist within the vaccination–IT-services and vaccination–utilities pairs. Moreover, the effect of vaccination on the Treasury bond index is negative, whereas the economic policy uncertainty shows an interchanging lead and lag relation with vaccination. It is further observed that the interrelation between vaccination and the corporate bond index is insignificant. Overall, the impact of vaccination on the sectoral equity markets and economic policy uncertainty is higher than on oil and corporate bond prices. The study offers several important implications for investors, government regulators, and policymakers.

## Introduction

The US economy and markets have been adversely affected by several systemic crises, including among the most severe, the Global Financial Crisis (GFC) and the ongoing COVID-19 pandemic (Crotty, 2009; Mishkin, 2011; Baker et al., 2020; Sharif et al., 2020; Albulescu, 2021; Arnold & Rhodes, 2021; Umar et al., 2021a). The Coronavirus originally detected in China in late 2019 has been rapidly transmitted to the US soon after, at the very beginning of 2020. As of October 03, 2021, the total numbers of Coronavirus cases, recoveries, and deaths in the US are, respectively, equal to 44.49 million, 33.92 million, and 0.72 million.^1^ The financial markets of the US have been hardly hit during the initial phase of the COVID-19 pandemic (Mazur et al., 2021; Qureshi, 2021; Yousaf et al., 2022a, 2022b). For instance, the S&P 500 index has lost one-third of its value from February 19, 2020, to March 23, 2020, the latter being the date of the apogee of the COVID-caused market meltdown, registered at the latter date. However, the arrival of the COVID vaccine at the end of 2020 has represented by far the biggest positive news for human beings and financial markets around the globe. Inspired by the above-mentioned works on the general influence of the COVID-19 progression/contention, herein we examine what is the specific impact that COVID-vaccination itself exercises over selected US markets during seven months since the beginning of the vaccination on December 20, 2020. Our study seeks further insights into the COVID-vaccine influence on the US financial markets.

COVID-vaccination has been playing an important role in the revival of the US economy and markets. The different variants of COVID-Vaccination have been introduced worldwide starting from December 2020 onwards. Currently, there are three vaccines authorized by the US government, namely Pfizer-BioNTech, Moderna, and Johnson & Johnson’s Janssen.^2^ Moghadas et al. (2021) measure the clinical effectiveness of COVID-vaccination in the US using 300-day-long data and report that vaccination has substantially mitigated the effects of the COVID-19 pandemic. This study documents that the attack rate was reduced from 9.0 to 4.6%, whereas the intense care unit (ICU) hospitalization, non-ICU hospitalization, and deaths were decreased by 65.6%, 63.5%, and 69.3%, respectively. Amidst many market-specific studies examining the influence of the COVID-19 pandemic on volatility of financial markets (Baek et al., 2020; Choi et al., 2020; Ahmad et al., 2021a, 2021b; Gherghina et al., 2021; Gubareva et al., 2021, 2022a; Laborda & Olmo 2021; Si et al., 2021; Yousaf & Yarovaya 2022; Mensi et al., 2022; Naeem et al., 2022, 2023; Arfaoui & Yousaf 2022; Hanif et al., 2023), we highlight the paper by Rouatbi et al. (2021), because it focuses specifically at the effects of the COVID-vaccination itself analyzing both, the developed and emerging stock market’s volatility using the data of 44 stock markets. This study find that COVID-vaccination positively contributes to the stability of global equity markets. Moreover, COVID-vaccination is found to be more influential for the developed than for the emerging equity markets. Further, Arfaoui et al. (2022) find that the connectedness between gold and energy markets varies over the pre and post COVID-vaccination periods. However, none of the above-mentioned studies have explored in detail the effect of the COVID-vaccination itself on the US financial markets. Therefore, we are motivated to address this literature gap.

We contribute to the existing literature by examining the connectedness of the COVID-vaccination with the economic policy uncertainty (EPU), oil, bonds, and sectoral equity markets in the US. Previously a large number of studies focused on examining the impact of the COVID-19 pandemic on the EPU (Choi, 2020; Albulescu, 2021; Dai et al., 2021; Yousfi et al., 2021; Dou et al., 2022; Al-Shboul et al., 2022), oil (Mensi et al., 2020; Sharif et al., 2020; Cao & Cheng, 2021; Gharib et al., 2021; Yousaf, 2021; Jiang et al., 2021a, 2021b; Atri et al., 2021), bonds (Andrieș et al., 2021; Arnold & Rhodes 2021; Gubareva 2021a, 2021b; Gubareva et al., 2021; Yi et al., 2021; Elsayed et al., 2022; Arif et al., 2022), and sectoral equity markets (Baek et al., 2020; He et al., 2020a, 2020b; Ahmad et al., 2021a, 2021b; Qureshi 2021; Shahzad et al., 2021; Umar et al., 2021a; Shahzad & Naifer, 2022). EPU in the US is measured using the daily data of US economic and policy-related news. Changes in EPU have important implications for asset pricing because higher uncertainty in economic policies leads to higher uncertainty in the firm’s future cash flows, which ultimately affects the stock prices and returns. The change in EPU is also important for policymakers and regulators because high EPU disrupts investment plans and consumption patterns in the economy (Baker et al., 2016). Hence, the relationship between COVID-vaccination and EPU is important to explore because it will ultimately provide important and useful information to the micro and macro-level stakeholders in the US.

The benchmark grade for US high-quality crude oil West Texas Intermediate **(**WTI). The WTI prices reached their lowest level, becoming negative (− $37) during the initial phase of the COVID-19 pandemic on April 20, 2020.^3^ The WTI touched their all-time lows because of two reasons: (a) a reduction in consumer spending on oil due to social distancing and lockdowns and (b) filled-up oil-storage capacity (Hansen, 2020; Corbet et al., 2020). It has been expected that with COVID-vaccination, the crude oil market would rise because of the foreseen increase in aggregate consumer demand in the US. The behavior of oil markets in the vaccination phase provides useful implications to the oil producers, oil-dependent firms, investors, and policy markets in terms of decision-marking for oil-production, portfolio allocation, hedging, firm level-budgeting, and overall stability of the market.

We also focus on the US corporate and government bond markets sensitivity to the COVID-vaccination. In the mid of March 2020 (initial phase of the COVID-19), the slowdown in businesses increased the bankruptcy risk which ultimately led towards the higher cost of borrowing and lesser accessibility of funds for the firms in the US (Nozawa & Qiu, 2021). However, in respect to the government bonds, Gubareva and Umar (2020) and Gupta et al. (2021) conclude the US Treasuries act as safe haven during the COVID-19 pandemic. In respect to the fixed-income domain, our study will examine whether safe haven features of US government bonds still remain in force after the beginning of COVID-vaccination. The vaccination-bonds nexus deserves further investigation in order to provide insightful information, potentially useful for investors, portfolio managers, monetary policy makers, and market regulators, allowing for sound decisions regarding investment opportunities, portfolio allocation, hedging, and fixed-income market stability.

The sectoral stock markets are highly sensitive to any uncertainty in economy, including the pandemic related uncertainty (Hoque & Zaidi, 2019; Haroon & Rizvi, 2020; Si et al., 2021; Umar et al., 2021c; Costa et al., 2022). During the initial phase of the COVID-19, the reaction of all US stock sectors was not same to the COVID-19. Mazur et al. (2021) find that the returns of food, natural gas, software, and healthcare stocks are positive, whereas the returns of real estate, petroleum, hospitality, and entertainment sectors fall dramatically during the COVID-19 triggered meltdown in March 2020. Goodell and Huynh (2020) report that during the COVID-19 in the US, pharmaceutical and medical product industries provide highly positive returns, whereas the returns of restaurants and hotels are substantially negative. The COVID-19 increases the business of those sectors whose demand of products and services is positively affected by the pandemic. For instance, the demand for health and medicine facilities increases with the rise in number of patients affected by Coronavirus. On the other hand, the restaurant and airline industries are affected due to lockdowns and bans on travelling in the country and across countries. Hence, in the COVID-vaccination phase, we can expect the recovery of the sectors of economic activity, severely damaged during the initial expansion of the COVID-19 disease. Therefore, in respect to the US stock market, our focus is to explore the per-sector performance of equities after the beginning of COVID-vaccination. This analysis provides valuable information potentially useful to investors, portfolio managers, and regulators regarding the portfolio allocation, hedging, and stock market stability in the US.

The findings of our study show the positive impact of COVID vaccination on the oil and sector indices over various frequency scales and periods, and the vaccination is perceived to lead the oil and sector indices. More specifically, we observe strong connectedness of vaccinations with communication services, financials, health care, industrials, information technology (IT) and real estate equity sectors. However, weak interactions exist within the vaccination–IT-services and vaccination–utilities pairs. Moreover, the effect of vaccination on the Treasury bond index is negative, whereas the economic policy uncertainty shows an interchanging lead and lag relation with vaccination. It is further observed that the interrelation between vaccination and the corporate bond index is insignificant. Overall, the impact of vaccination on the sectoral equity markets and economic policy uncertainty is higher than on oil and corporate bond prices. The study offers several important implications for investors, government regulators, and policymakers.

The rest of the paper is structured as follows: Sect. 2 provides the data and methodology, Sect. 3 presents the results, and Sect. 4 concludes the whole study.

## Data and methodology

### Data description

To examine the connectedness of COVID vaccination with oil, bonds, economic policy uncertainty, and the sectoral stocks in the US, this study makes use of daily data on COVID-19 vaccination, S&P 500 equity indices for 12 sectors of economic activity, US economic policy uncertainty (EPU), WTI (Crude Oil), S&P 500 corporate bond index and S&P Treasury bond current 10-year index. Following Sharif et al. (2020), the US EPU is measured using the daily data of US economic and policy related news, WTI benchmark crude oil quotes are used as proxy for oil prices, the US stock sectoral equity indices are measured by the S&P 500 sector indices. The original data series are converted into natural logarithmic series. The sample sectors are Consumer Discretionary, Consumer Staples, Communication Services, Energy (Oil and Gas), Financials, Health Care, Information and Technology (IT), IT Services, Industrials, Materials, Real Estate, and Utilities. The data on US COVID-19 vaccination is extracted from the websiteourworldindata.org.^4^ Moreover, the data on US S&P 500 corporate bond index and US S&P Treasury bond current 10-year index, and US S&P 500 sector indices are gathered from website of S&P Global.^5^ US EPU is sourced from the website of policyuncertainty.com. The data on crude oil (WTI) is taken from the website of US Energy Information Administration.^6^ The sample period is from December 20, 2020, to August 14, 2021, yielding 138 observations. The COVID-19 vaccination has started in the US from on December 20, 2020; therefore, we start sample period from this date.

### Methodology

#### Estimation technique: continuous wavelet transformation

The wavelet method is a powerful tool for investigating the association between variables across different investment horizons. The advantage of applying the wavelet method is that it allows tracking variables simultaneously at time and frequency scales, providing valuable insights into varying trends and interactions. The wavelet technique aids in determining the heterogeneous risk perceptions and expectations that prompt different reactions of investors, market analysts, and policymakers over investment prospects.

Additionally, to achieve detailed insights and estimation from daily data, the wavelet method is the suitable technique because the technique provides a better explanation of the short-run and long-run relationship between COVID vaccination, economic policy uncertainty, oil, bonds, and sectoral equity markets, which isolates elements that provide evidence across at various timescales. The reason for using daily data is based on its utility in assessing the impact of COVID-Vaccination policy on economic and market-specific variables since the subtleties of vaccination policy on economic variables can properly be determined using daily data. In addition, daily data aids in detecting short and long trends at various timescales; thus, the traditional methodologies may not be appropriate for achieving the objectives of the present study.

Moreover, unlike traditional time series techniques, the wavelets are effective in detecting lead and lag relations between nonlinear time series. In addition, the estimations from the traditional model unveil average analysis and generalization based on the complete sample. On the contrary, the wavelet models provide a detailed examination of the relationship between variables at diverse time scales. Besides, the wavelet method does not require assumptions of conventional data generation procedures. The wavelet analysis assists in uncovering the influence of the COVID vaccination policy on economic and market-specific factors that explain the lead and lag relationship, and aids in identifying visible and dominant trends and findings. The empirical findings are expected to define the role of vaccination policy in countering economic issues and volatility in important financial sectors of the US economy. Several studies apply the wavelet approach for the connectedness between various markets (Choi et al., 2020; Tiwari et al., 2019; Ali et al., 2022; Umar et al., 2021d; Gubareva et al., 2022b).

This study utilizes the continuous wavelet transform, designated as ${N}_{a}\left(p,q\right)$, which depicts the projection of a wavelet $\psi (.)$ opposite to the time sequence $a\left(t\right)$ ∈ ${k}^{2}$ (Ṟ), i.e.:

1 $$ N_{a} \left( {p,q} \right) = \int \limits_{{ - \infty }}^{\infty } a\left( t \right)\frac{1}{{\sqrt q }}\psi \left( {\frac{{\overline{{t - p}} }}{M}} \right)dt $$

The important characteristics of this technique lies in its power to decompose and reconstruct a time series $a\left(t\right)$ ∈ ${k}^{2}$ (Ṟ):

2 $$a\left(t\right)= \frac{1}{{C}_{\psi }}{\int }_{0}^{\infty }\left[{\int }_{-\infty }^{\infty }Na \left(p,q\right){\psi }_{p,q }\left(t\right)du\right]\frac{{d}_{q}}{{M}^{2}}, \quad M>0$$

In addition, this technique conserves the potential of the detected time series.

3 $$ \left\| {a} \right\|^{2}= \frac{1}{{C}_{\psi }}{\int }_{0}^{\infty }\left[{\int }_{-\infty }^{\infty }{\left|Na\left(p,q\right)\right|}^{2}dp\right]\frac{{d}_{q}}{{M}^{2}}$$

The current study considers the above-mentioned easy method in the shape of the wavelet coherence, which specifies the structure between two-time sequences in a bivariate archetypal format.

#### Wavelet coherence (WC)

Wavelet coherence (WC) is a supportive tool that measures the magnitude of correlation among the series considering the time-frequency space and distinguishes feasible relationships between the two time-series. Particularly, WC enhances the time correlation analysis by additionally taking into account a relevant frequency-scale information. In the present study, we use WC to examine the transmission effect between the COVID-19 vaccination, on one hand, and the selected economic indicators (EPU, oil, bonds) along with the sectoral equity indices, on the other hand. It is worth noting that WC has recently been gaining attention from academy, becoming widely applied among other time-series methodologies.

Torrence and Compo (1998) defined the cross-wavelet power and cross-wavelet transform (CWT) and explained that the CWT of the two time-series $a\left(t\right)$ and $b\left(t\right)$ can be specified as follows:

4 $${N}_{ab}\left(p,q\right)= {N}_{a}\left(p,q\right){N}_{b}^{*}\left(p,q\right)$$

where ${N}_{a}\left(p,q\right)$ and ${N}_{b}\left(p,q\right)$ stand for the two continuous individual transfigurations of the $a\left(t\right)$ and $b\left(t\right)$ time-series, *p* denotes the location index, *q* is the scale estimate, and symbol * denotes the complex conjugate. The CWT is used to compute cross-wavelet power by |${N}_{ab}\left(p,q\right)$|. The cross-wavelet power spectrum segregates the area in which significant cluster is unveiled in the time-frequency domain as compared to the time sequence under observation. The WC technique can assess the particular regions in the time-frequency space where unknown and important fluctuations occur in the time-frequency correlation of the time-series under consideration. Hence, we use the adjusted wavelet coherence equation as initially proposed by Torrence and Webster (1999):

5 $${W}^{2}(p,q)=\frac{{\left|M \left({M}^{-1}{N}_{ab} \right(p,q)\right|}^{2}}{{\left|M \left({M}^{-1}{N}_{a} \right(p,q)\right|}^{2}{\left|M \left({{M}^{-1}N}_{b}\right(p,q)\right|}^{2})}.$$

M here signifies smoothing operator. The squared wavelet coherence coefficient’s range is non-negative: 0≤ ${W}^{2}\left(p,q\right)$ ≤ 1. The proximity to zero indicates low tending to zero correlation, while values closed to 1 signify high correlation between the variables. The Monte Carlo method is applied to ascertain the WC’s hypothetical allocation.

## Empirical results and discussion

**Fig. 1:**
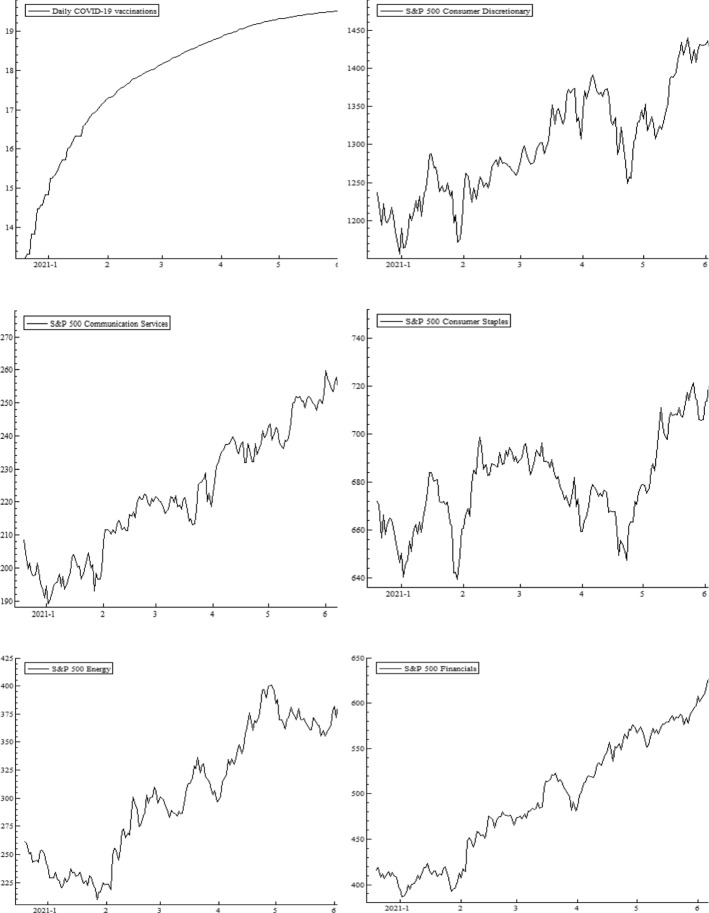

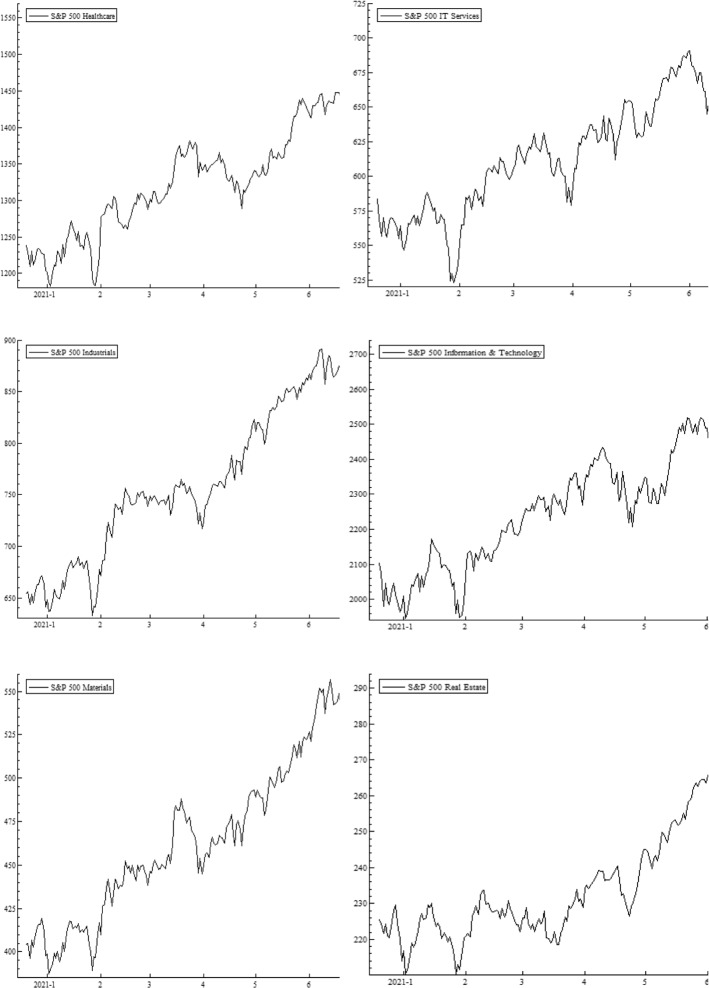

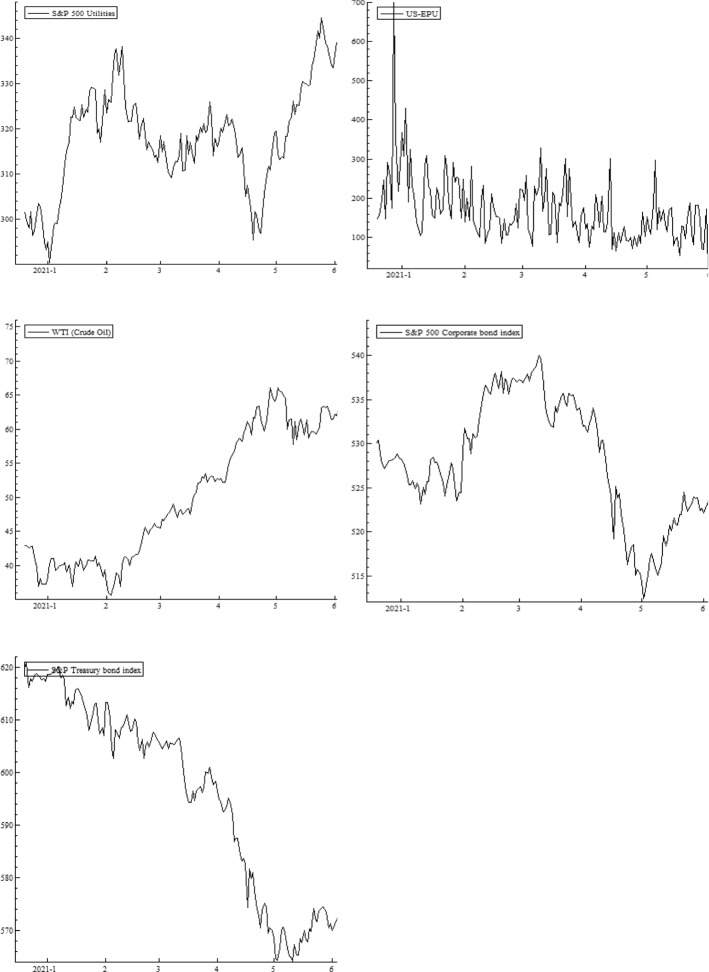
Evolution of sample variables. Plots of daily COVID-19 vaccination, S&P 500 sector indices, EPU, WTI, S&P 500 corporate bond index, and S&P Treasury bond current 10-year index. The sample period is from December 20, 2020, to August 14, 2021

Figure 1 presents the dynamic trends in daily data of log daily vaccinations, S&P 500 sector indices, USEPU, WTI (Crude Oil), S&P 500 corporate bond index and S&P Treasury bond current 10-year index. We notice a steady but saturating increasing trend in daily COVID-19 vaccination from for all the sample period. The sector equity indices also exhibit rising, however quite volatile trends. Further, US EPU is on its downward track, although it experiences severe volatility swings along the sample period. The daily time series plot for WTI reveals a rather persistent increase since February 2021 onwards. Moreover, the corporate bond and Treasury bond indices witness uneven drop down and then bouncing back patterns.

### Continuous wavelet transform

**Fig. 2:**
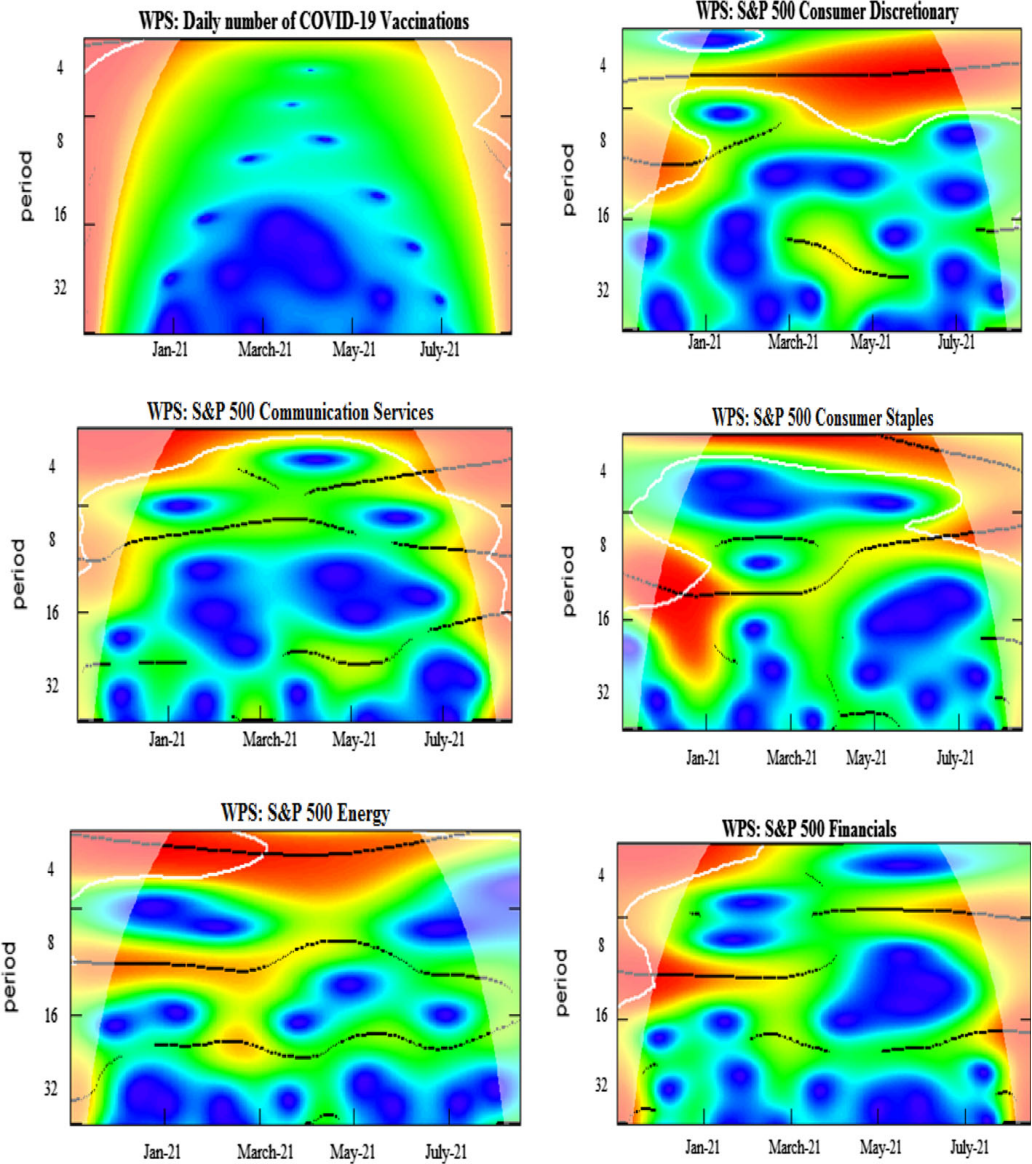

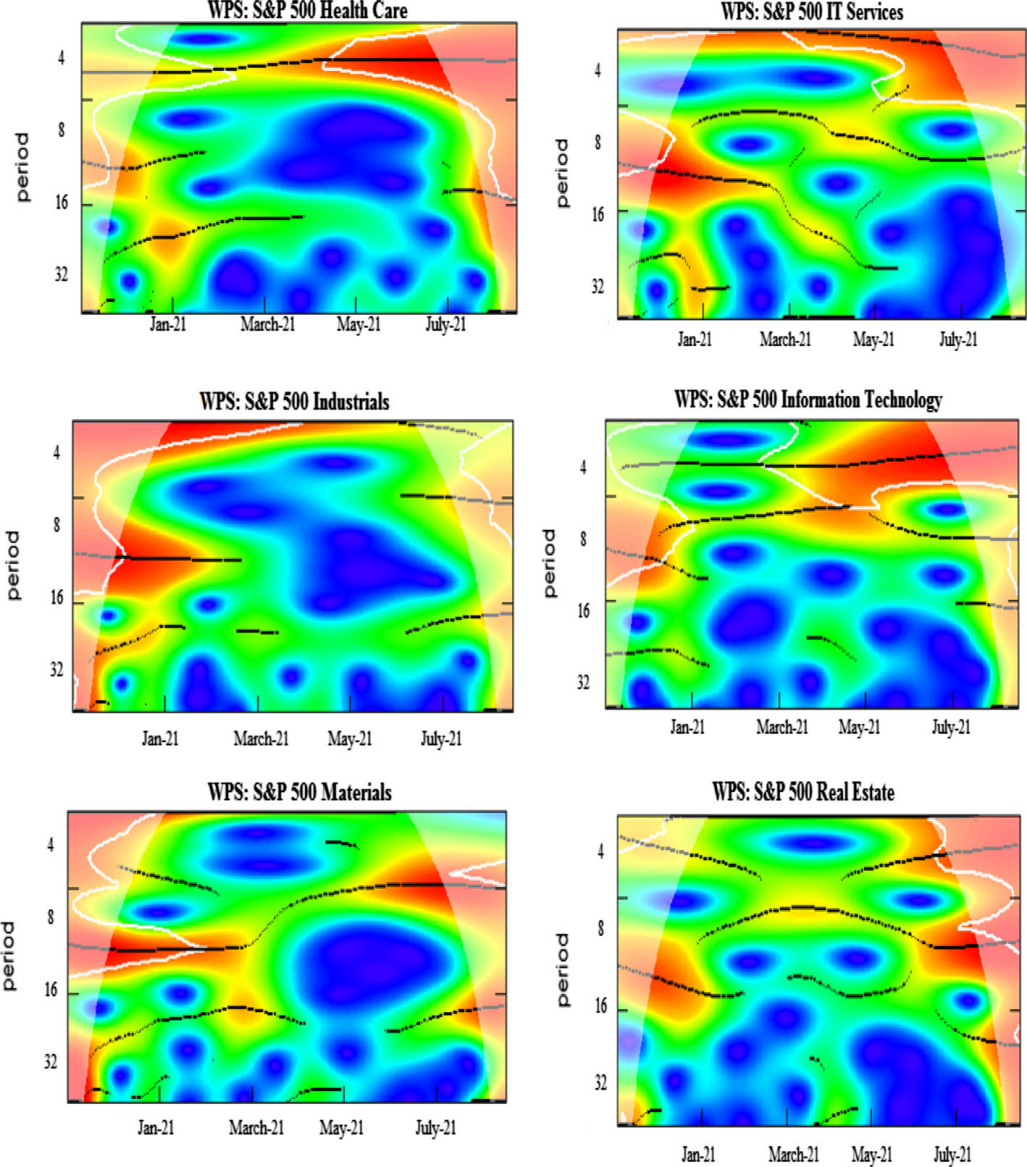

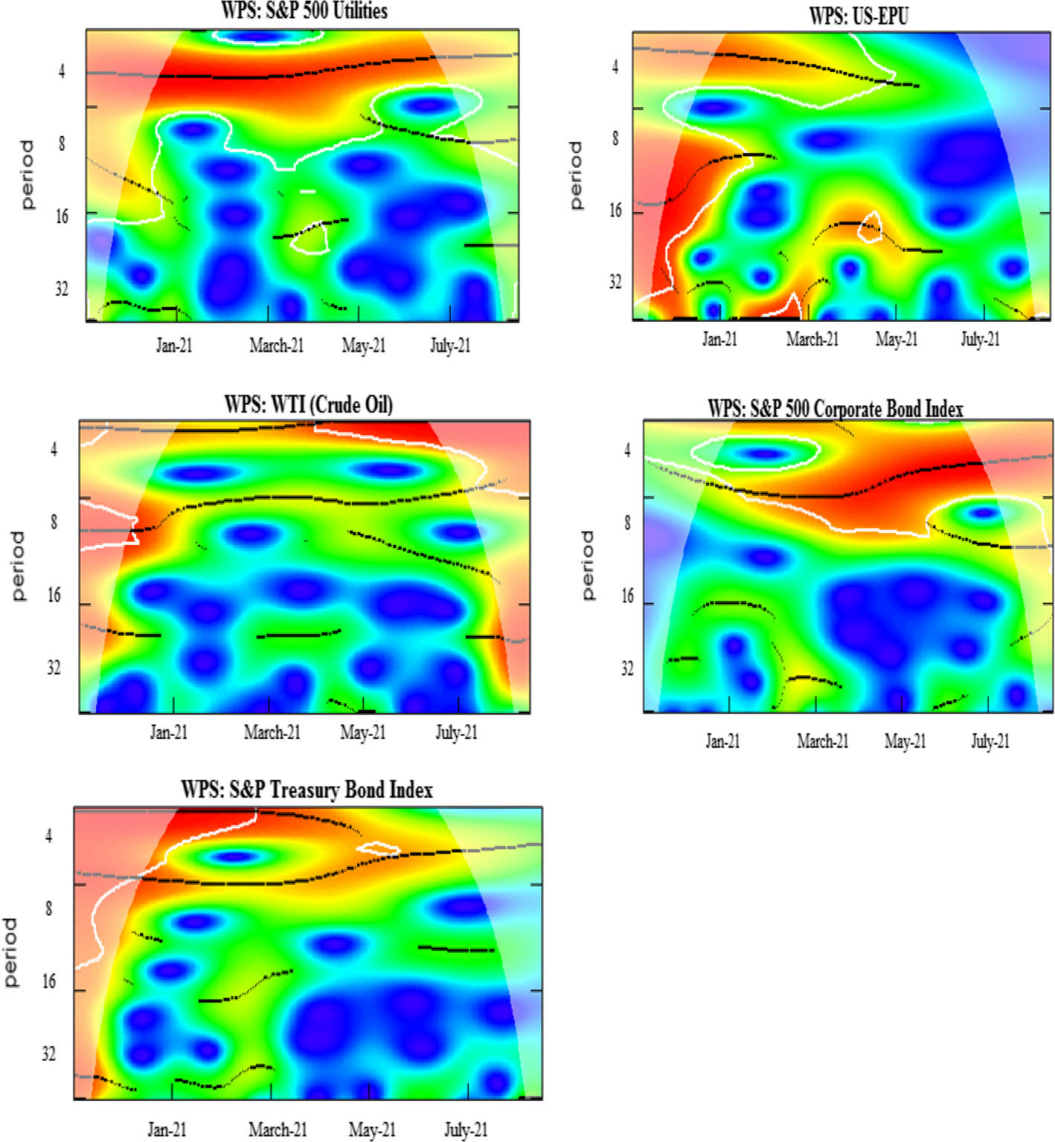
Wavelet power spectrum plots. Wavelet power spectra for COVID-19 vaccinations, S&P 500 sector indices, EPU, WTI,~ S&P 500 corporate bond index, and S&P Treasury bond current 10-year index. The horizontal axis represents the time and the vertical axis depicts the frequency scales. The black contours represent the significance level and the white lines indicate the cone of influence. The color ranges from blue to red for power intensity varying from low to high. (Color figure online)

The wavelet power spectrum (WPS) plots for each variable are illustrated in Fig. 2. The WPS shows the magnitude of variance in time and frequency domain. The horizontal axis represents the time and the vertical axis depicts the frequency scales. The statistical significance of the power spectrum is measured against null hypotheses of a stationary process through a background power spectrum. The estimations of Monte Carlo simulations utilize phase-based surrogate series, where the 5% level of significance is designated by the black contour. The cone of influence is exhibited by the white line. The power ranges from low to high intensities, depicted, respectively, by blue to red color tonalities.

The daily COVID-19 vaccinations exhibit temperate volatility in the series, as could be inferred from several blues spots with the turquoise aureoles in the central region of the respective panel. The US equity sectors show high levels of volatility at 4–8-day scale for different periods. The prominent power fluctuations along the vertical direction are witnessed through the intermittent interplay of reddish colors in the health care and IT sectors for a brief period around April 2021. The rationale behind short period of variations in these sectors may be attributed to the relative stability and slow-paced reactions to economic fluctuations along time (Ahmad et al., 2021a, 2021b). In addition, persistent volatility in energy, consumer discretionary, utilities, and industrials is evident from January to May 2021. The adverse effects may be attributable to different events in US during this particular time period, for example, losses incurred by investment funds and plunge in stock prices of GameStop (Umar et al., 2021b) that subsequently lead to restrictions in stock trading of several sectors. Another cause could be the detection in various states of news virus variants, such as Lineage P.1 and Lineage B.1.1.7. Moreover, among the BRICS and G7 countries, the US is most greatly affected by the pandemic with high infection destiny (Yu et al., 2021) and, in consequence, the pessimistic attitude of US investors triggers greater sensitivity in sector equity indices. Our findings corroborate the results of Ahmad et al. (2021a, 2021b), and Haroon and Rizvi (2020), who confirm the strong impact of coronavirus outbreak on industrials, financials, real estate and consumer discretionary sectors. Besides this, we also note a twist in volatility for consumer staples at 8-16-day frequency bands from the beginning of COVID-vaccination until January 2021, which disappears afterwards, implying that spread of vaccines in the subsequent months lead to lower volatility.

The EPU shows a highly volatile regime around December 2020 and January 2021, mostly at all frequency scales. However, the fluctuations tend to disappear thereafter, suggesting stability due to an increase in vaccination programs. Further, the existence of a scattered volatile region is noticed in WTI at 2–4-day high frequency. We tentatively attribute this short-run volatility to the price discounts by Saudi authorities to the US and main importers, leading to oil price wars and US economic policy uncertainty (Sharif et al., 2020). Overall, the volatilities are significant at high-frequency scales implying that the increase in vaccines and preventive measures induce decrease in fluctuations in the long run. Moreover, it is observed that red region for corporate bond index extends from February to August 2021. The new US government announces stimulus package in January 2021 followed by an instant raise in debt and borrowing rates. This period is characterized by high levels of health-related fear in US citizens driving increase in bond yields (Tzanakis, 2021). Similarly, the red region associated with volatility in Treasury bond index emerges from January and lasts until May 2021.

**Fig. 3:**
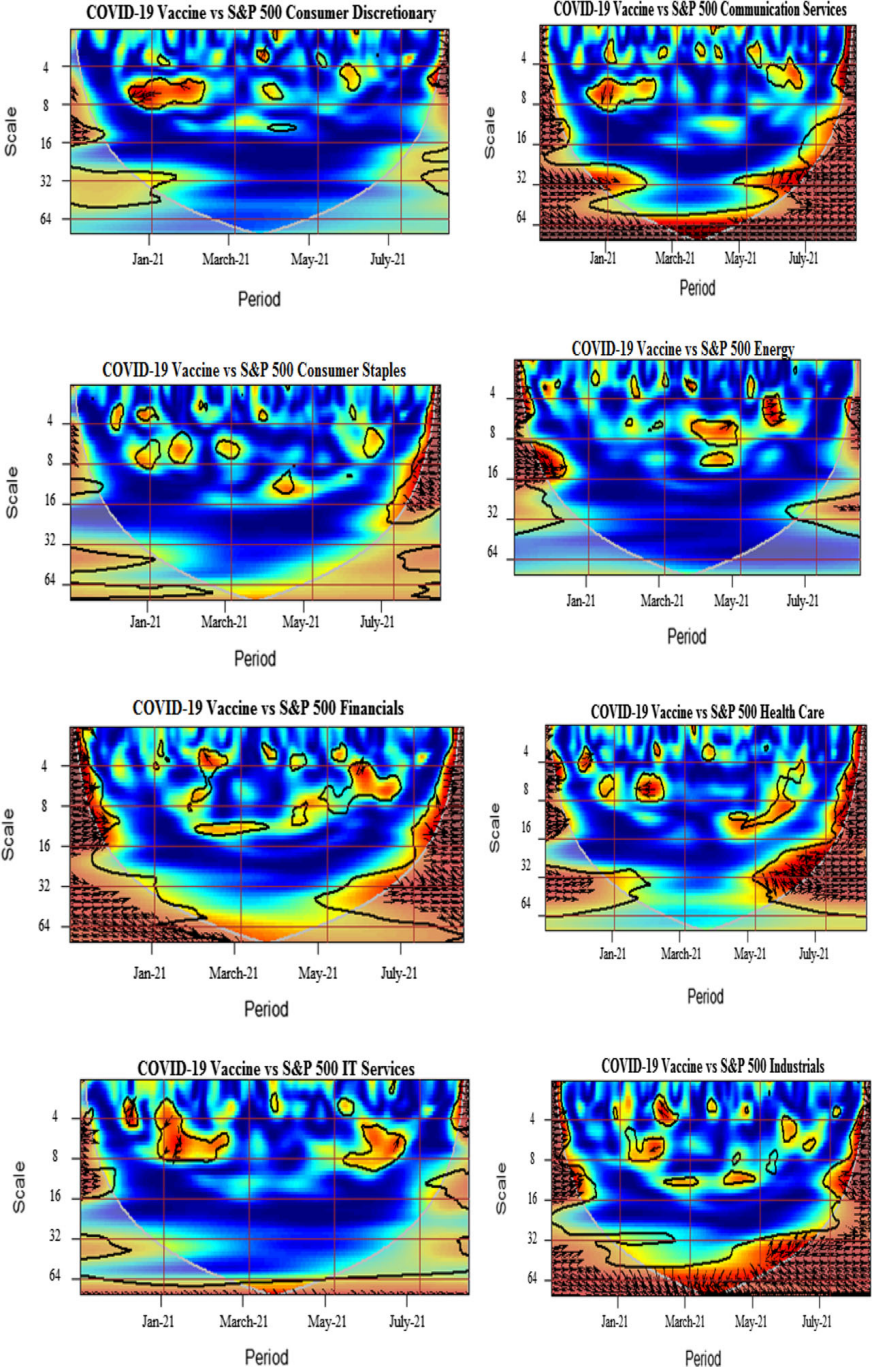

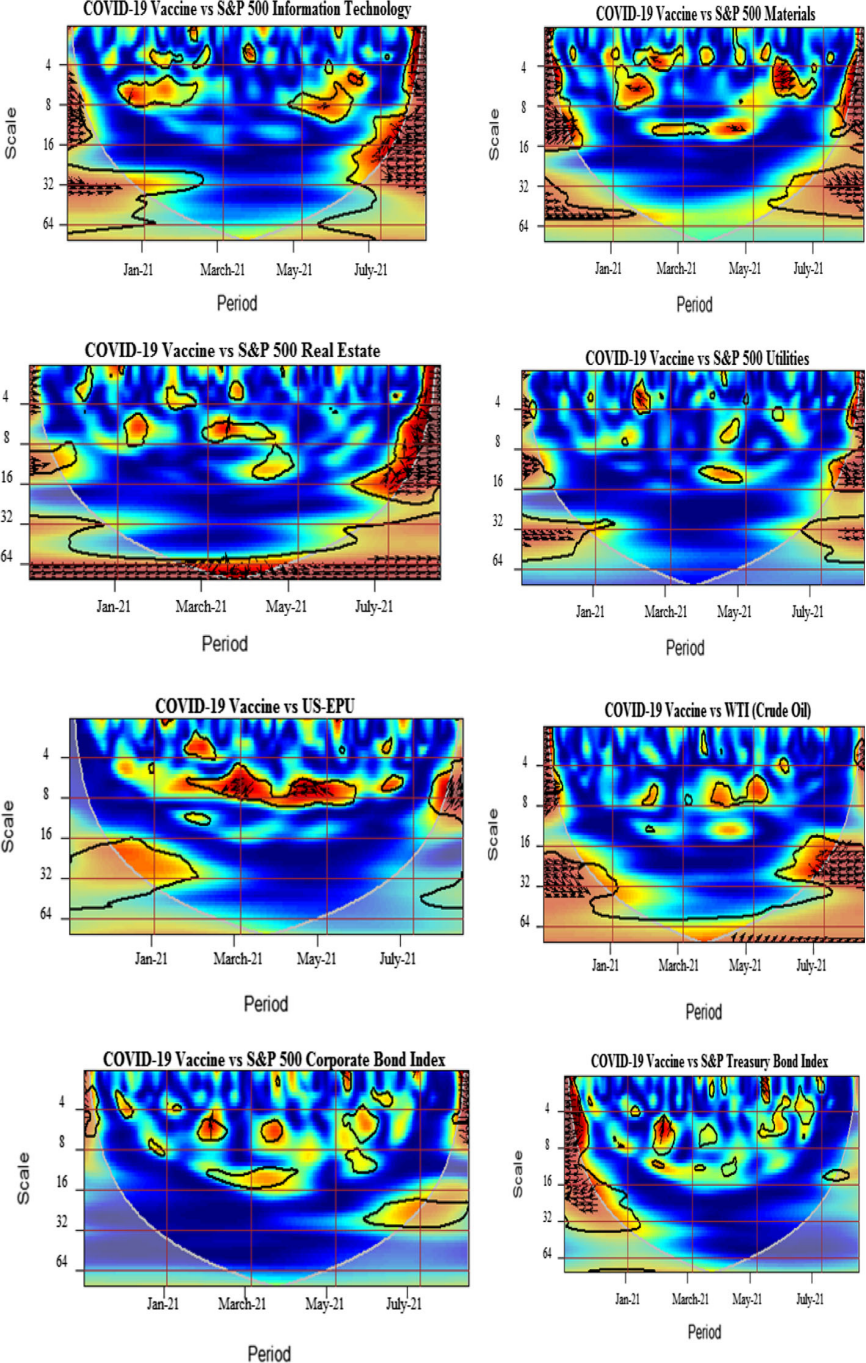
Wavelet coherence pairwise plots. Coherence plots between log daily COVID-19 vaccinations and S&P 500 sector indices, US EPU, WTI, S&P 500 corporate bond index and S&P Treasury bond current 10-year index. The black contour represents the 5% significance level and the white line indicates the cone of influence. The color tonality ranges from blue to red, respectively, for low to high coherence. The arrows pointing towards right depict the in-phase pairs, while the arrows pointing to the left indicate the anti-phase pairs. The right down or left up pointing arrows signify that the first series is leading, while the right up or left down pointing arrows indicate the lead by the second series. (Color figure online)

### Wavelet coherence

We further analyze the pairwise interactions between log of COVID-19 vaccination and other variables. Figure 3 presents wavelet coherence plots indicating the strength of correlations along with their extension in time-frequency space. The arrows in the coherence plots indicate phases. A zero-phase difference implies that the series moves together. The arrows pointing towards right (in-phase) indicate that the two time-series are positively correlated, whereas the arrows directing towards left (out of phase) signal of the negatively correlated time-series. More specifically, the in-phase pairs depict positive association, while the anti-phase pairs indicate negative association between the variables. The right down or left up pointing arrows signify that the first series is leading, while the right up or left down pointing arrows indicate the lead by the second series.

We note the existence of small islands of dependence at both higher and lower frequency bands over different periods for all vaccine–sector-index pairs. It is detected that significant co-movements between COVID-vaccination and consumer discretionary are manifested around January–February 2021 particularly at 4–8-day frequency scales. The plausible reason is that the implementation of the US vaccination programs in December 2020 resulted in the growth of business activities accelerating, in this manner, the recovery from health crisis-driven economic turmoil in early 2020 and causing increase in confidence in the US financial markets. The strengthened comovements during beginning of the sample are also attributable to the planning and stimulation of the COVID-vaccination in the US, especially taking into account the senate approved budget of $1.9 trillion for COVID-19 relief and authority announcements of 200 million doses of vaccines Pfizer and Moderna. However, in spite the increasing number of the administered vaccines and approval of various relief packages, the well pronounced comovements of the COVID-vaccination and sectoral equity indices, clearly observed at the beginning of the sample, weaken toward the middle of the analyzed period, indicating somewhat saturating marginal impacts of the already deaccelerated growth in vaccination levels. Amidst possible reasons of this effect, we indicate the termination of deal by the US government for supply of single antibody doses by Eli Lilly & Co., shortage in supply of vaccines and delays in vaccine orders due to increased demand, and the inefficaciousness of vaccines due to emanations of new variants (Forman et al., 2021; Jiang et al., 2021a, 2021b).The left heading arrows in the pairwise plot of the COVID-vaccination–consumer-discretionary coherence indicate an anti-phase behavior of these time series, probably due to the fact that the increase in the COVID-vaccination level is more beneficial for other than consumer discretionary sector, resulting in negative response of consumer discretionary stocks to initial rapid advancements in COVID-vaccination. The findings are in accordance with Kapar et al. (2022) who find negative response of consumer discretionary to announcement of vaccines in US.

Moreover, a high coherency region is identified for the vaccination–communication-services pair extending from May till July 2021 at around 1-month frequency. This may be interpreted imply that this sectors outperforms regaining market confidence with massive vaccine rollout. The findings are in line with the recent studies by Rouatbi et al. (2021), which conclude that COVID-19 vaccine plays an important role in stabilizing global equity markets, Yousaf et al. (2022a, 2022b), who find that media framing messages imprinting fear promote higher acceptance of vaccination.

We identify small and momentary coherences for consumer staples, materials, and energy sectors at the beginning and at the end of the sample period. These sectors exhibit slight effects of comovements at 4–16-day frequency scales. The arrows are predominantly pointing rightwards and down indicating aligned comovements with the COVID-vaccination leading the consumer staples and energy sectors. However, a mixed pattern is observed for the materials sector with interchanging lead and lag positions. The alternating coherence may be attributable to several factors influencing the equity dynamics in various sectors of economic activity. For instance, we highlight the strong spillover of oil price crisis to energy and materials sectors, documented by Ahmad et al. (2021a, 2021b), Laborda and Olmo (2021). The result is opposed to Demir et al. (2021) who find pronounced influence of vaccinations on energy stock volatility.

The coherence plot of the COVID-vaccination–financials pair shows tiny red color islands at the beginning and end of the sample period spread over 2–16-day frequency bands. The strengthened interdependence may be attributed to the reduced uncertainty and to the lower pandemic anxiety indices from the second half of 2020 onwards (Yu et al., 2021; Gherghina et al., 2021; Rouatbi et al., 2021). Further, we identify strong connectedness between health care and COVID-vaccination from May to July 2021. In the proximity to the right-hand border of the plot, we observe the vast red island, spreading over all frequency bands. The right pointing arrows indicate the predominance of the in-phase behavior of these two time-series. The findings support Kose et al. (2021), Mustapha et al. (2021), Biswas et al. 2021, and Turhan et al. (2022), who find that distrust in health care system leads to greater chances of vaccine hesitancy among public.

We find several islands of comovements for the COVID-vaccination–industrials pair. Strong correlations are seen at the beginning and at the end of the sample period at higher frequency bands. In addition, strong persistent coherence is witnessed from March to June 2021 at lower frequency bands, particularly at 32-day-plus scales. In particular, in June the right and upward directed arrows imply that of industrial sector time-series leads the COVID-vaccination time-series. As one of the possible plausible explanations could be mentioned a recognized contribution of industrials to systematic risk because of strong linkages of this sectors with other sectors of economic activity and oil prices (Laborda & Olmo, 2021). Moreover, the COVID-vaccination–IT pair exhibits several high coherence islands starting from May 2021 onwards, with in-phase relation as indicated by mostly right-headed arrows. The high coherence mapping is significant for a broadened periodicity from 2 to 32 days. The finding corroborates with Prescott and Prescott Jr. (2021) and He et al. (2021), who find that information technology applications, processes, and tools effectively increase immunization rate.

The coherence plot of the COVID-vaccination–real-estate pair reveals strong coherence from July 2021 onwards for the frequencies of 2–16 days. In addition, the significant positive connection is also manifested around the 64-day frequency from March to May 2021, where the vaccination is the leading variable. The strong positive connectedness is justifiable because of severe adverse impacts on real estate markets during the epidemic (Balemi et al., 2021).

Finally, the coherence maps for the COVID-vaccination–IT-services and COVID-vaccination–utilities pairs demonstrate a relative lack of mutual dependence throughout the sample period. This may be due to the fact that the US utility sector has shown stability over time throughout the episodes of pessimistic future market expectations (Ahmad et al., 2021a, 2021b). Moreover, the utility sector, although offering high mean returns since past decade, is less resilient to the business cycle and stock market depressions. Therefore, it is plausible that it appears less affected by the development and distribution of vaccines. The finding is consistent with Kapar et al. (2022) on the insignificant reaction of utilities sector to announcement of vaccines in US.

Wrapping up our analysis of the COVID-vaccination influence on the sectoral equity indices, we highlight the overall positive interrelations and leading role of the COVID-vaccination, suggesting that vaccine improves public health inclining towards a better pattern of public consumption, savings and investments (Masia et al., 2018). Indeed, as evidenced in the above presented analysis, the increasing COVID-vaccination results in uptrends in the sectoral equity indices. The findings are in line with Khalfaoui et al. (2021) indicating a positive influence of the COVID-vaccination on the S&P 500 returns.

The dependence structure between the COVID-vaccination and the US EPU reveals high coherence predominantly within the 5–10-day horizons for the period from February to July 2021. The arrows are turned left indicating an anti-phase behavior. They switch from mostly downward to mostly upward heading signaling that the US EPU lead during the initial months of the COVID-vaccination turns to the lead by COVID-vaccination over the US EPU time series during the most phase characterized by higher levels of the vaccinated population. However, the anti-phase behavior observed for all the high coherence region, imply that EPU decreases with increase in deployment of vaccination confirming positive signal for sectors and consumers impeding health crisis. This will eventually boost production, consumption, travel and education, investment and employment activities and overall global GDP. On the other side, the development and deployment of safe vaccines depends on economic policy interventions as high uncertainty may cause adverse effects on governments, corporations, and households, which pose challenges to the health care system in achieving herd immunity. These our findings to some extent corroborate with Hasan et al. (2021), documenting negative impact of COVID-19 cases on global economic activities. Along similar lines, Altig et al. (2020) emphasize the time required for vaccinations deployment as a riskier element for economic certainty put in perils by the pandemic.

The COVID-vaccination–WTI interconnection analyzed in the respective plot shows the presence of the pronounced red zone within the 16–32-day frequency band during July 2021. The arrows pointed to the right indicate an in-phase rather synchronized behavior. The plausible reason for simultaneous increases in COVID-vaccination and WTI is due to the severe adverse impact, which the initial escalation of the pandemic has produced on oil prices and world energy index (Sharif et al., 2020; Hasan et al., 2021).

Further, for the COVID-vaccination–Treasury-bond-index pair, the high connectedness corresponds to 2–8-day frequency horizons at the very beginning of the sample period in December 2020. In addition, a small isolated red island is seen in February 2021 within the 4–8-day frequency band. We associate its appearance with the announcement of the stimulus packages by the new US government (Tzanakis, 2021) that has positively affected the Treasury bond index putting it on the uptrend coinciding with the already observed uptrend in the COVID-vaccination. Further, the US 10-year Treasury bond yields showed an increase of 1.675% after announcement of vaccine since March 2020 indicating economic recovery (Kapar et al., 2022).

Furthermore, the relationship between vaccination and corporate bond index is negligible due to the colder regions at higher and lower frequency scales. The finding corroborates with Tzanakis (2021) who finds that subordinated bond yields are not correlated to Treasury yield changes and COVID-19 cases, being the stimulus package the key factor driving subordinated yield changes. The finding contradicts with To et al. (2022) who report that the vaccination news mitigates volatility in bond markets.

Overall, our findings evidence that the COVID-vaccination affects sectoral equity indices more strongly than the bond indices and the WTI. The results presented above confirm strong connectedness of the COVID-vaccination advances with communication services, financials, health care, industrials, IT and real estate sectors of economic activity. However, the IT services and utilities are the least affected. The findings reveal that the hardest hit sectors during pandemic (Industrials, Financials, Energy and Real estate) positively benefit from the vaccines. The investor sentiment hypotheses and asset pricing perspective claim that stock markets are expected to respond positively and immediately to vaccine announcements (Martins, 2022). The plausible explanations on sectoral connectedness may be enumerated correspondingly. First, since the message content of media manifests less fear supporting vaccinations (Yousaf et al., 2022a, 2022b), thus, media strategies deliver effective campaigns for raising public awareness regarding vaccines. Messages framing risk awareness, vulnerability and safety benefits are the most effective communication strategy in combating hesitancy of vaccine among public. Besides, various information technology tools create awareness regarding the health benefits of vaccination. Scheduling vaccine appointments through mobile apps, patient portal and websites entail technology comfort. Tele-health services and the Facebook global surveys provide useful insights regarding behavioral norms and patterns of the respondents regarding vaccinations. Furthermore, Information technology has formulated coordinated efforts in vaccine development programs with several institutions across the globe, for example, US Food and Drug Administration, US National Institute of Health and WHO (Talukdar et al., 2021).

Second, the health care sector specially biotechnology and research and development subsector show significant reaction to vaccine rollout. This is because the vaccine hesitancy undermines success of vaccination programs. Therefore, strong health care system is critical to address challenges for example, vaccine myths, mistrust in pharmaceutical companies and health experts (Biswas et al., 2021). Moreover, the clinical recommendations influence vaccine rates and reduce safety concerns consequently improving uptake of vaccinations.

Third, the performance of manufacturing industry was devastated during pandemic due to cessation of supply for imported raw materials. Supply chain disruptions, lower sales and higher fixed and production costs adversely affected industry cash flows. The sales dropped for residential and commercial properties and households confronted payment difficulties in redeeming mortgages (Balemi et al., 2021). Consequently, the easing of restrictions and opening of economic activities impacted the industries favourably.

In addition, moderate connections are documented within the COVID-vaccination–consumer-discretionary and COVID-vaccination–consumer-discretionary pairs. This is due to the fact that hotels recorded substantial gains during COVID-19 pandemic while entertainment sub-sector lost (Kapar et al., 2022). Moreover, the COVID-vaccination–EPU pair also unveils the strong interdependency, suggesting long term negative effect between variables. The results illuminate that the spread of vaccines reduce uncertainty increasing demand and boosts market optimism anticipating shrinkage in restrictions.

### Robustness check

**Fig. 4:**
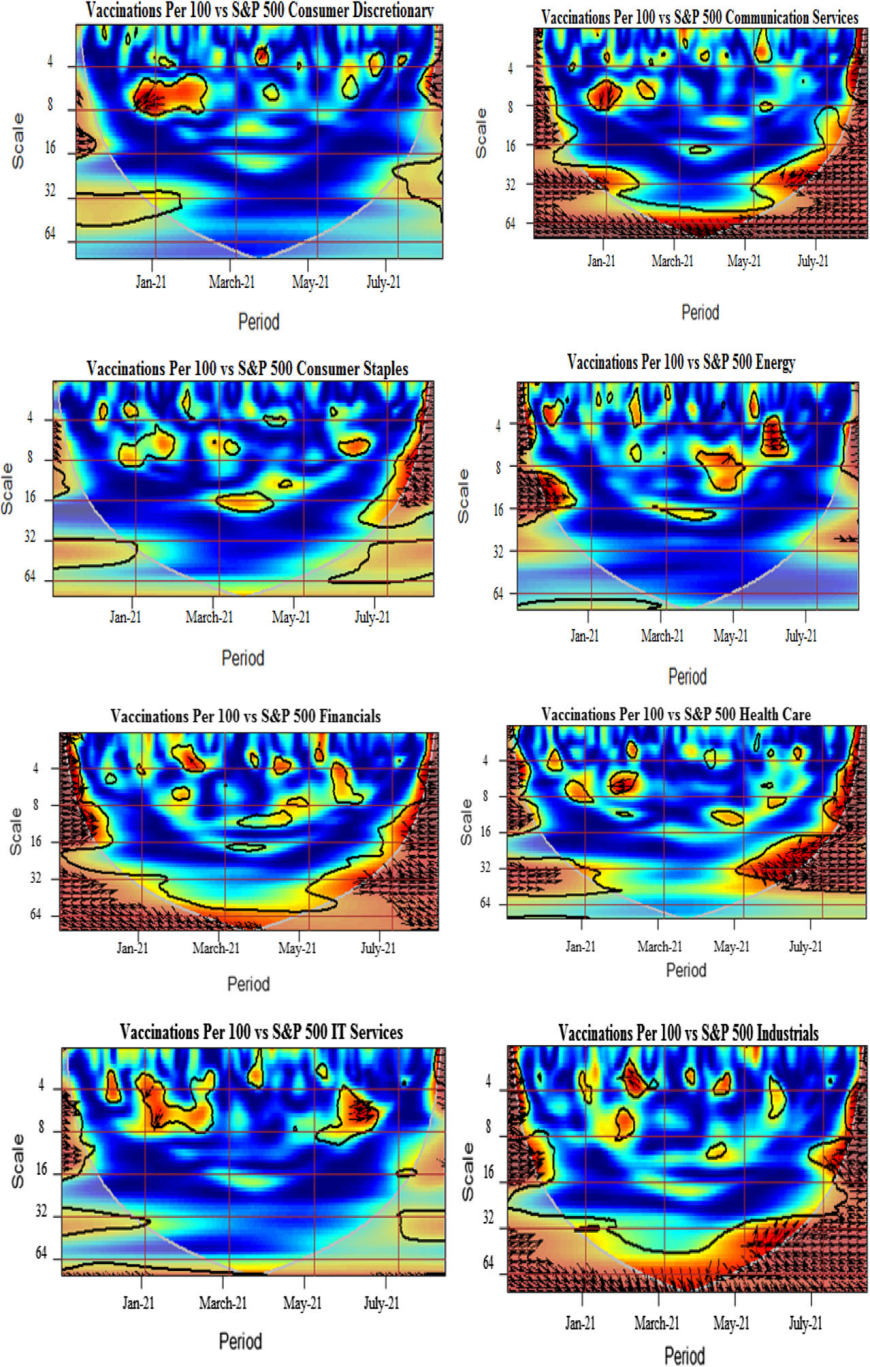

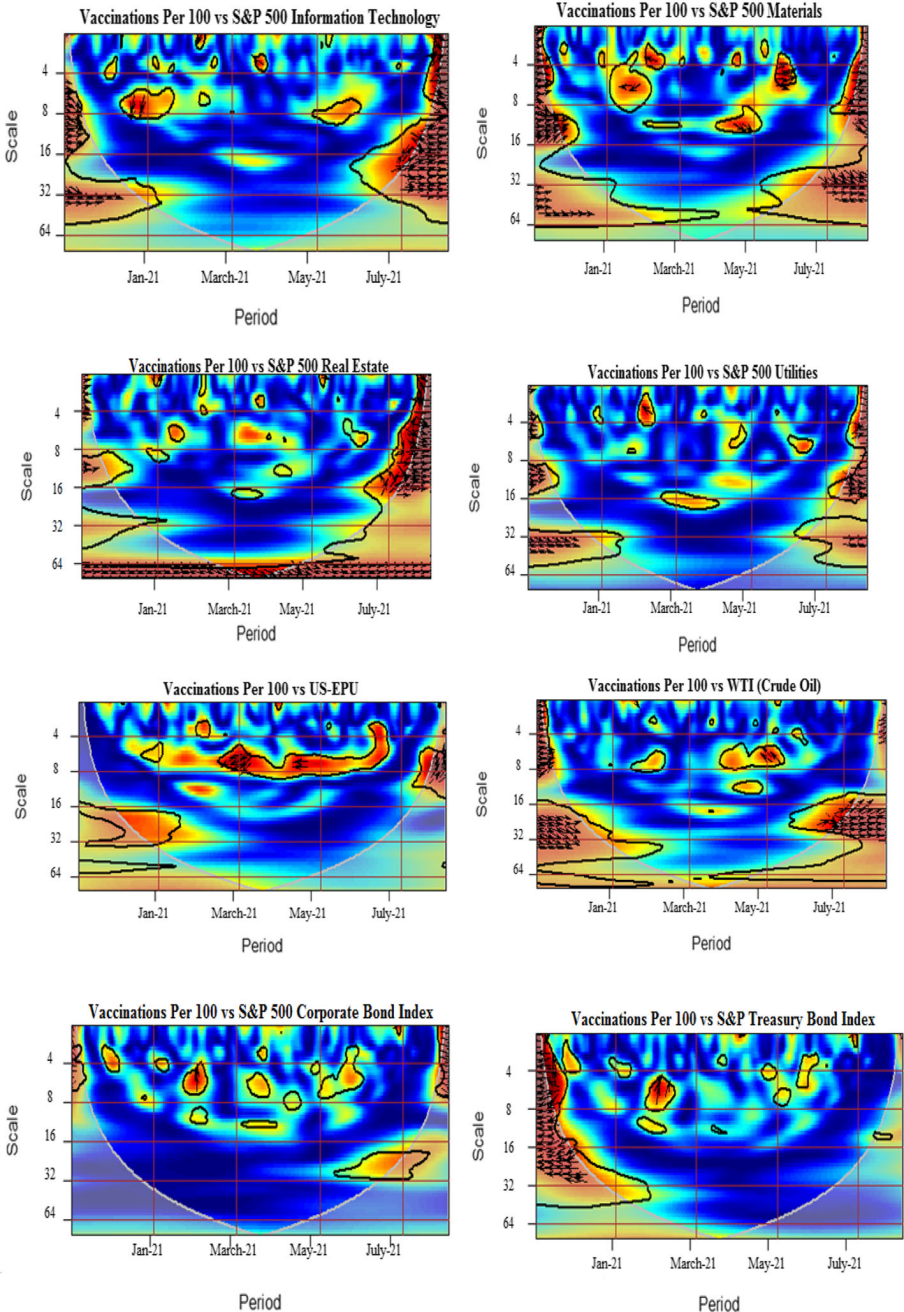
Wavelet coherence pairwise plots (robustness check). Coherence plots between daily COVID-19 vaccinations per 100 and S&P 500 sector indices, US EPU, WTI, S&P 500 corporate bond index and S&P Treasury bond current 10-year index. The black contour represents the 5% significance level, and the white line indicates the cone of influence. The color tonality ranges from blue to red, respectively, for low to high coherence. The arrows pointing towards right depict the in-phase pairs, while the arrows pointing to the left indicate the anti-phase pairs. The right down or left up pointing arrows signify that the first series is leading, while the right up or left down pointing arrows indicate the lead by the second series. (Color figure online)

To ensure the validity of our findings, we use an alternative measure of COVID vaccination that is daily vaccinations per one hundred (100) people. Figure 4 shows the wavelet coherence plots for each pair. The baseline findings are consistent with the results obtained using the measure of daily vaccinations per 100. We find the consistent positive co-movements between the vaccination and sectoral indices indicating significant short run effects for most of the pairs. The vaccination per 100 is found to lead most of the sector indices. Consequently, the investors may benefit with increase in number of vaccinations in the US. The findings are expected as the strategy against the pandemic causes the economy and the financial market recovery (Khalfaoui et al., 2021). Further, the negative relation with the US EPU from March to June 2021 at higher frequency horizons is substantiated. The arrows signify both lead and lag influences over the other. Our results also confirm that lower policy uncertainty signals successful execution of vaccination programs. We see that the WTI is positively influenced by the COVID-vaccination indicating optimistic trading and economic conditions. The WTI is found to lag behind the COVID-vaccination. This relationship is remarkable during the end of the sample within the 16–32-day frequency band. Consequently, investors may devise their portfolios while considering the vaccination policies and the lengths of horizons. Moreover, our supplementary analysis confirms the negative and insignificant impact of the COVID-vaccination on the Treasury bond index and corporate bond index, respectively, implying in these two cases the relative relevance of several other factors apart of the pandemics, influencing bond markets. E.g., we highlight forward looking expectations, liquidity dynamics, the US presidential election, stimulus packages, and Secondary Market Corporate Credit Facility announcements, among many other.

## Conclusion and policy implications

Our study examines from multi-horizon perspectives, the effect of the US daily COVID vaccination on sectoral equity markets, economic policy uncertainty, crude oil, government bonds, and corporate bonds. The empirical evidence is presented using wavelet-based approach, which analyzes interactive time-frequency connectedness. We employ the recent US daily data from December 20, 2020, to August 14, 2021.

Our findings unveil that the sectoral equity indices are mostly sensitive at high frequency scales, indicating substantial impact of short-term variations while suggesting the absence of long-term coherence, even in spite of increase in number of vaccinations. The corporate bond index exhibits high persistent volatility. In addition, the wavelet coherence findings indicate positive associations between the daily vaccination rates and the sectoral equity indices. More specifically, the COVID-vaccination is strongly connected with the communication services, financials, health care, industrial, information technology and real estate sectors, whereas weakly connected with the IT services and utilities sectors. Moreover, the COVID-vaccination positively leads the sectoral equity indices, implying that the COVID-vaccination plays a positive role in the recovery of equity sectors.

We observe positive connectedness between the COVID-vaccination and the crude oil market, with the former leading the latter. We evidence that oil markets perform well after the start of the COVID-vaccination in the US. Furthermore, the economic policy uncertainty is negatively associated with the COVID-vaccination, indicating that the vaccination exercises a positive influence on the US economy and markets, by means of reducing the level of aggregate uncertainty. Treasury bond (corporate bond) market is negatively (insignificantly) associated with the COVID-vaccination. Overall, the COVID-vaccination has a greater effect on sectoral equity indices in comparison to crude oil and bond indices. The vaccination-EPU interrelation exhibits interchanging lead and lag effects on each other. The robustness test using alternative measure of the COVID-vaccination,—namely, daily vaccinations per 100,—confirms the baseline analysis.

Our findings provide practical implications for the US government regulators and policymakers in transitioning back to economic normality. The different reactions of the analyzed herein sectors of economic activity to the COVID-vaccination provide significant implications to investors, possessing portfolio exposures to different industries. The investors may opt for portfolio adjustment for the strongly and moderately connected sectors. More precisely, the support packages may be provided to the sectors that are negatively affected due to vaccine rollout. The sectoral connectedness implies that strategic use of communication services is crucial to support vaccine campaigns. Further, the strengthened government policies through health protocols shall be prioritized. Similarly, the health information technology should be focused by providing in-person assistance and engaging local community partners. And the public health agencies may conduct webinars to reduce lack of confidence in vaccines. Further, the time and frequency connectedness of relevant sectors may also guide policy makers in designing macroprudential policies taking into consideration an additional dimension of investment horizons. Moreover, the significant and bidirectional effect of the COVID-vaccination and EPU suggests prioritization of vaccination programs for reinforcing economic returns. Thus, designing expansionary and coherent policies strategy for effective immunization is imperative. In addition, the government should intervene to overcome the vaccination related challenges for long run economic recovery. The positive dynamics between the COVID-vaccination and crude oil suggests that the massive spread of vaccination positively impacts the oil price. The finding is of concern to energy sector and traders. The private investors and companies may fund investments with lower cost of capital encouraging stable economic development. The negative and leading impact of Treasury bond index over the COVID-vaccination suggests that the vaccination programs may be executed in accordance with anticipation of the policies set by the US Treasury department. Finally, the stimulus packages should also be reassessed to deal with the intense risks in the corporate bond markets.

For the future studies, we recommend estimating the impact on the COVID-vaccination on the other global financial markets including ESG, green markets, precious metals, and agriculture commodities, etc., Further, we apply the wavelet approach, however, the impact of COVID-vaccination on markets can be examined using the event study methodology and quantile connectedness approach, etc., Finally, we suggest investigating the impact of the COVID-19 on markets using the high frequency data.

## Footnotes

1 https://www.worldometers.info/coronavirus/country/us/.

2 https://www.cdc.gov/coronavirus/2019-ncov/vaccines/different-vaccines.html.

3 https://www.bbc.com/news/business-52350082.

4 https://ourworldindata.org/covid-vaccinations.

5 https://www.spglobal.com/spdji/en/.

6 https://www.eia.gov/dnav/pet/hist/rwtcD.htm.
